# Comparative Transcriptome Analysis Reveals Relationship among mRNAs, lncRNAs, and circRNAs of Slow Transit Constipation

**DOI:** 10.1155/2021/6672899

**Published:** 2021-08-23

**Authors:** Shuai Yan, Yinzi Yue, Mingming Sun, Yinghui Chen, Xiaopeng Wang, Haihua Qian

**Affiliations:** ^1^Department of Anorectal Surgery, Suzhou TCM Hospital Affiliated to Nanjing University of Chinese Medicine, Suzhou 215009, China; ^2^First Clinical Medical College, Nanjing University of Chinese Medicine, Nanjing 210023, China; ^3^Department of Anorectal Surgery, Affiliated Hospital of Nanjing University of Chinese Medicine, Nanjing 210029, China

## Abstract

**Background:**

Slow transit constipation (STC) is characterized by persistent, infrequent, or incomplete defecation. Systematic analyses of mRNA, lncRNA, and circRNA expression profiling in STC provide insights to understand the molecular mechanisms of STC pathogenesis. The present study is aimed at observing the interaction of mRNAs, lncRNAs, and circRNAs by RNA sequencing *in vivo* of STC.

**Methods:**

A rat model of STC was induced by loperamide. The expression profiles of both mRNAs and miRNAs were performed by RNA sequencing. Enrichment analyses of anomalous expressed mRNAs, lncRNAs, and circRNAs were performed in order to identify the related biological functions and pathologic pathways through the Gene Ontology (GO) database and Kyoto Encyclopedia of Genes and Genomes (KEGG) database.

**Results:**

In total, 26435 mRNAs, 5703 lncRNAs, and 7708 circRNAs differentially expressed were identified between the two groups. The analyses of GO and KEGG show that (1) upregulated genes were enriched in a positive regulation of GTPase activity, cell migration, and protein binding and lipid binding and (2) GO annotations revealed that most *trans*-target mRNAs are involved in the regulation process of immune signal together with the proliferation and differentiation of immune cells. Additionally, the protein-protein interaction (PPI) network of differentially expressed (DE) mRNAs was constructed. Interestingly, all of the core lncRNAs and their coexpression mRNAs in this network are downregulated. Moreover, downregulated circRNAs have a set of target mRNAs related to immunoreaction, which was consistent with the overall tendency.

**Conclusion:**

Our investigation enriches the STC transcriptome database and provides a preliminary exploration of novel candidate genes and avenues expression profiles *in vivo*. The dysregulation of mRNAs, lncRNAs, and circRNAs might contribute to the pathological processes during STC.

## 1. Introduction

Slow transit constipation (STC) is one of the refractory digestive tract diseases. Commonly, this syndrome concludes slow colonic peristalsis and delayed excretion of intestinal contents, except normal rectal discharge and normal pelvic floor function, which is the most common subtype of functional constipation [1]. Reference investigation shows that the incidence of chronic constipation is increasing, even though it has become one of the key factors that affect people's quality of life globally. The epidemiological survey of chronic constipation shows that the incidence of chronic constipation is 9.9% in southern China, besides the prevalence rate significantly increases with age [2]. According to the statistical data of Europe and the United States, the incidence of constipation may be higher than expected. At least 65% of constipation patients are treated with laxatives on their own [3]. Patients with chronic constipation often have progressive difficulty in defecation, abdominal distension, sometimes they will gain introverted personality even depression. The above syndrome seriously declines life quality and could lead to complications (i.e., myocardial infarction, stroke, and colorectal cancer) [4]. Meanwhile, patients with intractable constipation show obvious psychological problems, which are often accompanied by anxiety, paranoia, obsessive-compulsive disorder and social maladjustment. It affects life quality and physical and mental health; with these, it causes serious burden to the social economy [5].

Current increasing studies have shown that STC has numerous pathogenesis and complex mechanism. Therefore, it is difficult to fully clarify the pathogenesis from one facet. Additionally, the clinical treatment is not sufficient [6, 7]. Long noncoding RNAs (lncRNAs), a little understood type of transcribed RNA molecules, have over 200 nucleotides in length and no significant protein-coding capacity, which have been identified as the key regulators of various biological functions [8, 9]. circRNAs are a genetic element, which are evolutionarily conserved and covalently closed. Some of them are rich in eukaryotes possessing cell-specific and tissue-specific expression profiles [10].

To disclose complex and heterogenous mechanisms of STC, we used a rat model of STC induced by loperamide and simultaneously performed mRNA, lncRNA, and circRNA microarray analyses to identify mRNA, lncRNA, and circRNA interactions, plus explored how these interactions influence on the pathogenesis of STC.

## 2. Materials

### 2.1. Drugs and Reagents

Loperamide hydrochloride and 4% paraformaldehyde were obtained from Sigma-Aldrich Co., Ltd. (St. Louis, MO, USA). Enzyme-linked immunosorbent assay (ELISA) kits of gastrin (GAS), motilin (MTL), substance P (SP), and 5-hydroxytryptamine (5-HT) were purchased from (Biolegend, San Diego, USA). The anti-C-kit antibody and AQP3 primary antibody were purchased from Proteintech Company (IL, USA). Microcentrifuge tubes, charcoal powder, and TRIzol reagent were obtained from Nanjing Institute of Built Bioengineering, Ltd. (Nanjing, China).

### 2.2. Animals

Male Sprague-Dawley rats aged 6-9 weeks were purchased from the Jiangsu Laboratory Animal Centre in Suzhou, China (License approval No.: SCXK [Su] 2017-0007). They were fed at a room temperature of 26°C with 45%-55% of the humidity and 12 h light/dark cycles. The environment was clean and quiet (low noise level ≤ 60 dB) in a well-ventilated place. All experiments involved in this study were performed under the requirements of the Provision and General Recommendation of the Chinese Laboratory Association. The study was approved by the Medical Research Committee on Animal Care and Use of Suzhou TCM Hospital Affiliated to Nanjing University of Chinese Medicine.

### 2.3. Methods

#### 2.3.1. Loperamide-Induced Constipation Model

In this study, animals were randomized into two groups: the control group (*n* = 5) and Lop group (*n* = 5). Rats were given normal saline in the control group, while the Lop group were induced with 4 mg/kg loperamide (an antidiarrheal drug) suspension to make the STC models (oral administration, twice per day: 09:00 and 17:00) for 14 days [11].

#### 2.3.2. Parameters Evaluated

Fecal pellets of rats were collected after treatment of 24 h to collect their total number, stool weight, and water content. All measurements were performed five times.

#### 2.3.3. Measuring Intestinal Charcoal Transit Ratio

The intestinal motility by charcoal meal was assessed following Kim et al. [12]. Briefly, on day 14, all rats were fasted for 12 h but no limited water, after which, they were fed charcoal within 10% acacia gum. 0.5 hours later, the rats' intestines from pylorus to ileocecal junction were removed; then, the total length of the truncated intestine and the charcoal transport distance was measured. Finally, the rate of intestinal motility was calculated using formula (1). Charcoal transit

(1) $$\begin{array}{c} \text{ratio }\left(\%\right)=\frac{\text{Distance traversed by the charcoal }\left(\text{cm}\right)}{\text{ Total length of small intestine }\left(\text{cm}\right)} \end{array}$$

#### 2.3.4. Measuring the Serum Concentration of Neurotransmitter

The blood of 5-10 mL was collected from the abdominal aorta and injected into the tube of EDTA anticoagulant. Then, it was separated 2 mL of plasma by centrifugation (3000 r/min, 30 min) to detect indexes of gastrin (GAS), motilin (MTL), substance P (SP), and 5-hydroxytryptamine (5-HT) by commercial ELISA kits (Biolegend, San Diego, USA). The procedure was strictly performed according to the kit instructions.

#### 2.3.5. Histopathological and Immunohistochemical Analyses

Colon tissue samples were collected from the sacrificed Sprague-Dawley rats. They were fixed at room temperature with 10% buffered formalin for 48 hours. The fixed colonic tissues were embedded in paraffin before being sliced into 5 *μ*m thick sections. Then, the slices were deparaffinized and stained using hematoxylin and eosin (HE; Sigma-Aldrich Co.). After that, we analyze their histological morphology and the thickness of mucosa and muscle with the Leica Application Suite (Leica Microsystems, Switzerland). C-kit proto-oncogene protein (C-kit) and aquaporin 3 (AQP3) expression levels were analyzed with immunohistochemistry (IHC). Then, the paraffin colon sections of rats were routinely fix with 4% paraformaldehyde for 10 minutes; after rinsing with phosphate-buffered saline for 3 times (3 minutes per time), 50 *μ*L of peroxidase blocking solution dropwise was added and incubated at room temperature for 10 minutes. Then, the slides were incubated consecutively overnight at 4°C with anti-CD117 (1 : 1000) and AQP3 (1 : 1000) primary antibody and at room temperature with HRP-conjugated anti-rabbit IgG incubation for 30 minutes. They were then stained using diaminobenzidine (DAB). By applying the high-power microscope (×200) and Image-Pro Plus 6 graphic processing software, the expression of C-kit and AQP3 per unit colon tissue area was observed and analyzed. Each section was observed with 5 fields randomly. The optical density values of C-kit and AQP3 expression of rats in both groups were statistically and, respectively, assessed.

### 2.4. Statistical Analysis

All data were expressed as the mean ± standard deviation. They were subjected to a one-way analysis of variance (ANOVA) and Dunnett's *t*-test. *P* values less than 0.05 indicated significant or very remarkable differences which were marked with ^∗^.

### 2.5. RNA Extraction, Library Preparation, and Sequencing

Total RNA extracts were obtained following the manufacturer's instructions by TRIzol®. The clear supernatants containing the total RNA extracts were transferred to fresh tubes and stored at −80°C. A total of 3 *μ*g RNA was used for each sample as input material. The RNA-Seq library was generated using NEBNext® Ultra™ Directional RNA Library Prep Kit for Illumina® (NEB, USA) following the manufacturer's instructions. Finally, the Illumina HiSeq 4000 system was used to sequence all the libraries and generate 150 bp paired-end reads.

### 2.6. Quality Control, Alignment, and Quantification of RNA-seq Data

Raw data of fastq were processed through in-house perl scripts to get the clean data. Reads with adapters or low quality were removed, and Q20, Q30, and GC contents of the clean data were calculated to evaluate the quality of sequencing.

Paired clean reads were mapped to the rat reference genome (rn6) with HISAT2 (v2.1.0). The transcript of each sample was assembled from the mapped reads by StringTie (v1.3.3) based on annotated transcript file from ENSEMBL. For unannotated transcripts (novel transcripts), we used CNCI (Coding-Non-Coding-Index) (v2), Pfam Scan (v1.3), and CPAT (Coding Potential Assessment Tool) (v1.2.4) to predict coding potential. Novel mRNAs or lncRNAs were defined while the three above tools simultaneously reported with or without coding potential. Subsequently, FPKMs (fragments per kilobase of transcript per million mapped fragments) of both mRNAs and lncRNAs were calculated by StringTie (v1.3.3).

When identifying circRNA, CIRCexplorer (v2.2.3) was used to find circularizing junction and spliced sequence with the fusion junctions obtained from TopHat2. Candidates with junction reads ≥ 2 were considered bonafide circRNAs, and the expression levels of circRNAs were estimated by TPM (transcript per million).

### 2.7. Identification of Differentially Expressed Transcripts

The mRNAs (DE mRNAs) and lncRNAs (DE lncRNAs) expressed differentially between the STC and non-STC groups were identified by DESeq2, and the different expressions of circRNAs (DE circRNAs) were identified using the limma package in R. log_2_ fold change | ≥1 and *P* value < 0.05 were indicated.

### 2.8. Protein-Protein Interaction (PPI) Network Construction

The STRING database (v 11.0) was used to predict the potential interactions among proteins translated of top 300 DE mRNAs (|log_2_ fold change|rank), confidence score ≥ 0.7 were selected. Visualization of PPI network was achieved through Cytoscape software (v3.8.0, http://www.cytoscape.org/).

### 2.9. Analysis of Target Genes Regulated by lncRNAs

The lncRNA function is mostly achieved by acting on target genes in *cis* or *trans*. *cis*-acting elements were DNA sequences that are adjacent to the structural portion of a gene that regulates gene expression. Hence, we selected DE mRNAs within 100 k upstream or downstream of DE lncRNAs. *trans*-acting factors usually are proteins binding to *cis*-acting elements to control gene expressions. Therefore, a coexpression network of lncRNA-mRNA was constructed according to the interregulatory correlation between DE lncRNAs and DE mRNAs. The Pearson correlation coefficient (PCC) was calculated using log_2_(FPKMs + 1), when ∣PCC | ≥0.98 and *P* value < 0.001 were considered meaningful. The network was visualized by Cytoscape.

### 2.10. circRNAs-miRNAs-mRNAs Network Construction

circRNA-miRNA-mRNA interactions were predicted by miRanda (v3.3a). The upregulated and downregulated circRNAs/mRNAs were selected to construct a ceRNA network by Cytoscape software.

### 2.11. Functional Enrichment Analysis

The enrichments of Gene Ontology (GO) and Kyoto Encyclopedia of Genes and Genomes (KEGG) pathways were performed for the host genes of DE mRNAs by using DAVID (v6.8) and KOBAS (v3.0) software, respectively. *P* < 0.05 was recognized significant.

## 3. Results

### 3.1. Effect of Loperamide on Fecal Pellets

**Figure 1:**
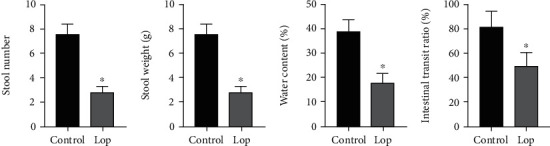
Alterations of stool number (a), stool weight (b), water content (c), and intestinal transit rate (d) *in vivo*. Data represent the mean ± standard deviation from five replicates. ^∗^*P* < 0.05 versus the control group.

The STC rat model was established by loperamide inducing. The total number and weight of feces were remarkable lower in the Lop group than the control group (Figures 1(a) and 1(b)). In addition, fecal water content was decreased more than the Lop group (Figure 1(c)). The outcomes of fecal parameter changes indicate that the in vivo model was built (Figure 1).

### 3.2. Effect of Loperamide on Intestinal Transit Rate

The intestinal propelling movement of carbon ink displayed that the intestinal transit rate in the Lop group was remarkably lower than that in the control group (Figure 1(d)).

### 3.3. Effects of Loperamide on Neurotransmitter Concentration

**Figure 2:**
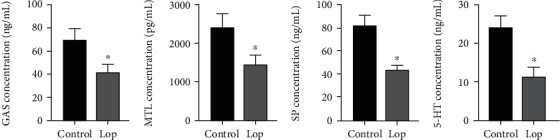
Gastrointestinal hormone levels in loperamide-induced constipated rats. Data represent the mean ± standard deviation from five replicates. ^∗^*P* < 0.05 versus the control group. GAS: gastrin, MTL: motilin, SP: substance P, and 5-HT: 5-hydroxytryptamine.

Commonly, constipation would lead to colonic motor dysfunctions, intestinal neurological abnormalities, and disease states. The combination of myogenic, intestinal plexus, and extrinsic neurons could affect colonic motor activity [12]. Gas, MTL, SP, and 5-HT play an important role in regulating gastrointestinal motility. Loperamide can significantly reduce more Gas, MTL, SP, and 5-HT than the control group (Figures 2(a)–2(d)).

### 3.4. Histopathology and Immunohistochemistry Findings

**Figure 3:**
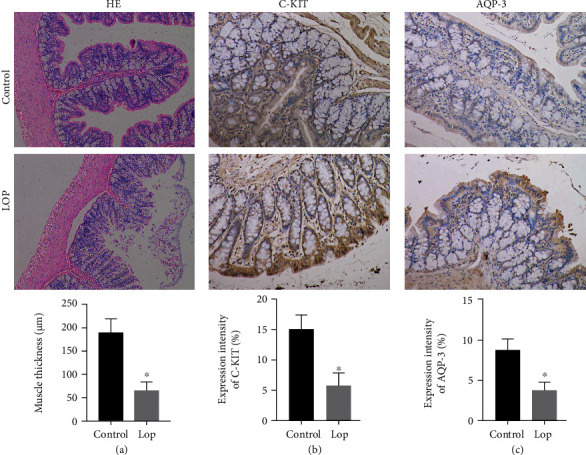
HE staining results for colon sections in rats of the control group and the Lop group (Figure 3(a)). C-kit and AQP3 protein expressions at the colon site in the control and Lop groups were measured by immunohistochemical staining (Figures 3(b) and 3(c)). Data represent the mean ± standard deviation from five replicates. ^∗^*P* < 0.05 versus the control group.

The pathological changes in the colon tissue were observed in the Lop group, with damage to colonic mucosa, thickening of the submucosal muscle, and the decrease in intestinal water (Figure 3). A group of cells, which exists in all layers of the colon [13] and interstitial cells of Cajal (ICC), plays an important role in regulating intestinal motility. C-kit could maintain the normal phenotype of ICCs during development and maturation [14]. AQPs have recently been observed to have an integral effect on human water transport systems [15]. In the human colon, AQP3 has been demonstrated to be important to colonic water transport, and it is mainly expressed in mucosal epithelial cells [16]. In the control group, C-kit- and AQP3-positive immunoreactive structures are dyed in brown. The expressions of C-kit and AQP3 decreased more in the Lop group than the control group.

### 3.5. Quality Test and Alignment Analysis of RNA-seq Data

The clean data of 9 samples were obtained with an average of 56.63 million reads and 16.99G bases per sample (Table 1). The Q30 ratio was >98%, and no GC bias was observed, suggesting sequencing clean data are qualified. Over 94% of the clean reads were perfectly mapped to the rat reference genome (rn6), and 67.83-77.50% uniquely mapped reads were obtained from the total reads from the 9 samples, which indicated an excellent performance of the sequencing reads with high credibility of the results in downstream analysis.

### 3.6. Characterization of Transcripts

**Figure 4:**
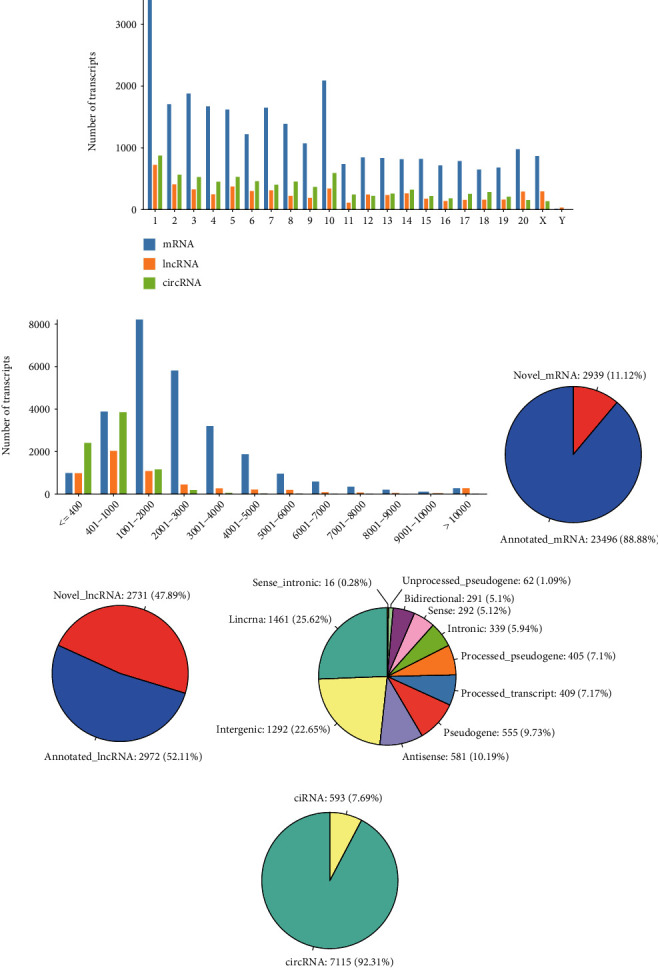
Characterization of mRNAs, lncRNAs, and circRNA. (a, b) Chromosomal (a) and length (b) distribution of mRNAs, lncRNAs, and circRNAs. (c, d) Proportion of novel transcripts for mRNAs (c) and lncRNAs (d). (e, f) Types of the identified lncRNAs (e) and circRNAs (f).

After assembly, a total of 26435 mRNAs, 5703 lncRNAs, and 7708 circRNAs were identified in STC rats among 9 samples. Overall, the chromosomal distribution of mRNAs, lncRNAs, and circRNAs were consistent. Most transcripts were mainly located on the first 10 chromosomes (Figure 4(a)), in which the larger chromosomes contained more lncRNAs. The transcript lengths of mRNAs were longer than those of noncoding RNAs, especially the length greater than 1000 nt. 80% of the total number of noncoding RNAs was no longer than 2000 nt, while 60% of them were approximately 200 to 2000 nt in length (Figure 4(b)). The amount of novel lncRNAs accounted for nearly half proportion of mRNAs (Figures 4(c) and 4(d)), which suggested that there were still many unknown regions in the rat genome. We also classified noncoding RNAs that were identified in this study and the numbers and proportions of different types for lncRNAs (Figure 4(e)) and circRNAs (Figure 4(f)).

### 3.7. Differential Transcription Expression Profile Analyses

**Figure 5:**
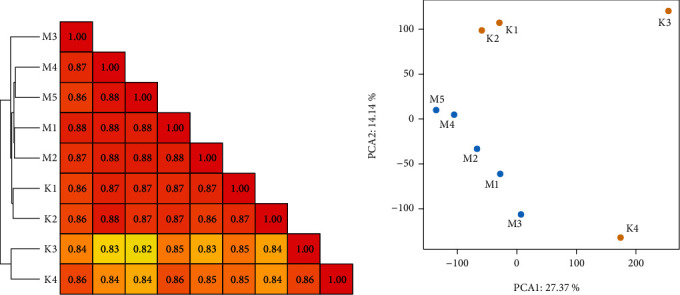
Relationship analysis between the STC and non-STC transcriptome samples. (a) Hierarchical clustering heat map of transcriptomic expression data; the color scale indicates the degree of the Pearson correlation. (b) The level of correlation or differentiation among samples shown by the principal component analysis (PCA) plot.

Correlation coefficient and principal component analysis (PCA) of all transcripts could represent the degree of similarity between groups. The analysis results showed that within group were highly correlated, while between groups were clearly distinguished (Figure 5).

**Figure 6:**
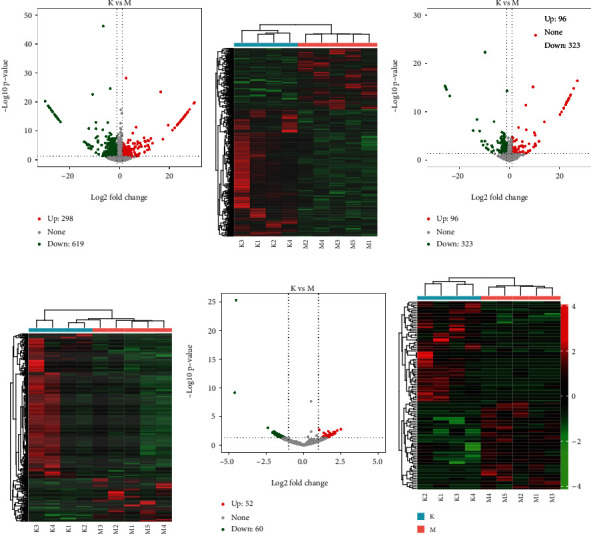
Analysis of differentially expressed mRNAs, lncRNAs, and circRNAs. (A, c, e) Volcano plot of DE mRNAs (a), DE lncRNAs (c), and DE circRNAs (e). Red and green, respectively, indicate the upregulated and downregulated expressions. (b, d, f) Heat map shows the expression profile of DE mRNAs (b), DE lncRNAs (d), and DE circRNAs (f) among each sample. The horizontal line in the volcano map represented the fold (log2 scaled) down or up changes, the vertical line represented a corrected *P* value of 0.05 (-log10 scaled), green spots indicated the differentially downexpressed RNAs with statistical significance, red spots indicated the differentially upexpressed RNAs with statistical significance, and light grey spots indicated RNAs with no statistically significant expression.

To identify the different expression of mRNAs (DE mRNAs), lncRNAs (DE lncRNAs), and circRNAs (DE circRNAs), *P* ≤ 0.05 and ∣log2 fold change | ≥1 were used as the threshold. Volcano plot showed a set of significantly differentially expressed transcripts were aptly delimited both in the STC and non-STC groups (Figures 6(a), 6(c), and 6(e)). A total of 917 DE mRNAs were determined, including 298 upregulated and 619 downregulated mRNAs. DE lncRNA analysis indicated 419 deregulated lncRNAs, among those, significantly upregulated and downregulated lncRNAs were 96 and 323, respectively. For circRNAs, out of 112 deregulated, 52 upregulated and 60 downregulated were observed. Unsupervised cluster analysis of the DE mRNAs, DE lncRNAs, and DE circRNAs also showed an obvious expression patterns between two groups (Figures 6(b), 6(d), and 6(f)). All differentially expressed transcripts were listed in Table S1.

### 3.8. GO and KEGG Enrichment of DE mRNAs

**Figure 7:**
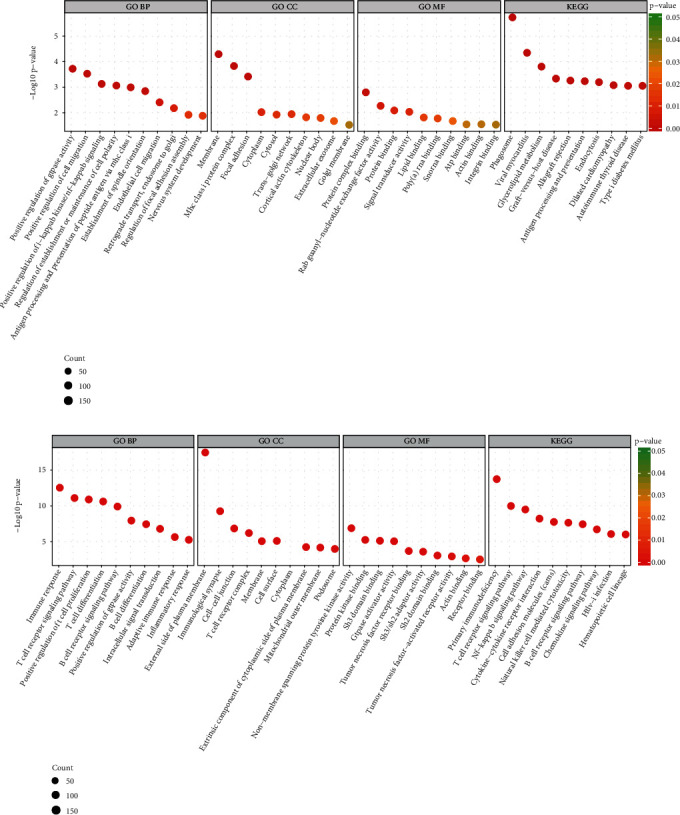
The GO and KEGG pathway annotations of differentially expressed mRNAs. Only the top 10 most significantly enriched terms from biological process (BP), cellular component (CC), molecular function (MF), and KEGG categories were listed. The color corresponds to the significance, and the size indicates the number of genes enriched in each term.

We performed GO and KEGG enrichment analyses for the host genes of DE mRNAs to predict the potential biological function of mRNAs.  Figure 7 shows the top 10 enriched terms for each category. Upregulated mRNAs were mostly enriched: biological process (BP) that was related to positive regulation of GTPase activity and cell migration, in terms of cellular component (CC) related to the membrane and cytoplasm, and molecular function (MF) related to protein binding and lipid binding. Significantly enriched signaling pathways were involved in phagosome and lycerolipid metabolism (Figure 7(a)). For downregulated mRNAs, several terms directly related to the immune system were consistently observed, such as immune response and inflammatory response in BP, immunological synapse and T cell receptor complex in CC, and T cell receptor signaling pathway and NF-kappa B signaling pathway (Figure 7(b)). Functions associated with the immunoreaction, cell movement, and energy metabolism were significantly changed, which indicated that the activation or inhibition of related gene expressions might accelerate the pathological process.

### 3.9. Protein-Protein Interaction (PPI) Network of DE mRNAs

**Figure 8:**
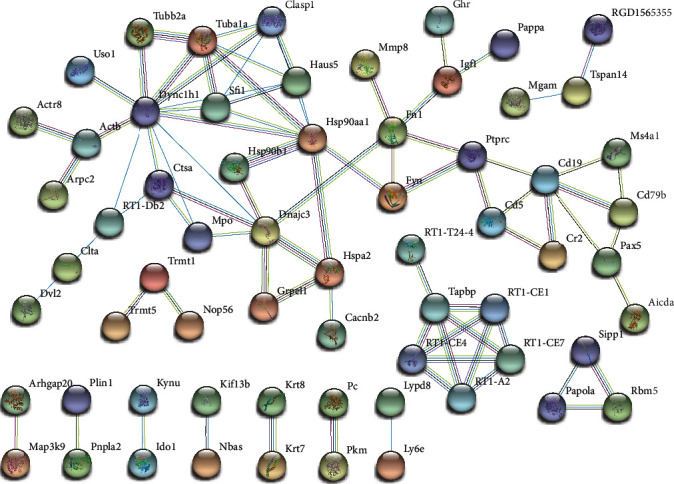
STRING network visualizing the functional protein association in STC using top 300 DE mRNAs. Nodes in the network represent proteins and the color of edges indicate known or predicted interactions.

To investigate the important role of protein interactions in STC rats, a PPI network analysis were performed using the STRING based on the top 300 DE mRNAs (Figure 8). We found that 64 proteins formed a complex functional network, and proteins which had high connectivity with other proteins, including Dync1h1 (degree = 13), Cd19 (degree = 9), Ptprc (degree = 9), Hsp90aa1 (degree = 9), Dync1i2 (degree = 8), Ccr7 (degree = 8), and Fn1 (degree = 8) were identified hub proteins. Hub proteins were widely involved in important cell cycle or immune-related pathways (Table 2), which indicated that dysregulation in one of these proteins can have significant effects on STC.

### 3.10. Functional Identification of DE lncRNAs

Previous studies reported that lncRNAs *cis*-regulated neighboring and overlapping genes, or coexpression with their target genes by *trans*-factors [17, 18]. To elucidate the potential function of lncRNAs, DE mRNAs within a 100 kb of upstream or downstream in the DE lncRNAs were considered *cis*-target, while targets in *trans* were predicted via calculating the expressed correlation.

**Figure 9:**
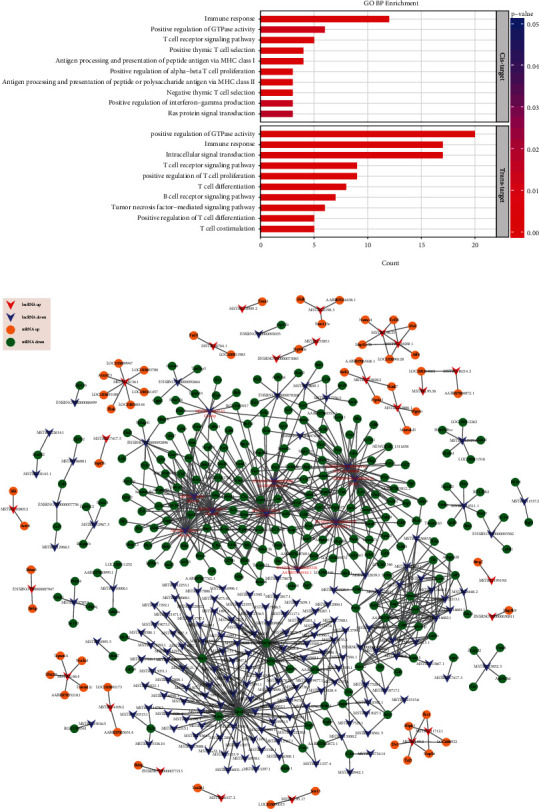
Functional identification of DE lncRNAs. (a) Gene enrichment analysis for the host genes of DE mRNAs as *cis*- or *trans*-target for DE lncRNAs. Top 10 GO terms of biological process are shown, *x*-axis denotes the number of genes overlapping with each term, and color corresponds to the significance. (b) Coexpression analysis for interaction between DE lncRNAs and DE mRNAs. V-shaped nodes and ellipse represented lncRNA and mRNA, respectively. The node size represented the corrected *P* value (larger nodes for more signifcant *P* values). Read and purple colors represented upregulated and downregulated lncRNAs, respectively. Orange and green colors represented upregulated and downregulated mRNAs, respectively.

A total of 109 possible *cis*-regulatory relationships were identified (Table S2). Among them, the proportion of downregulated lncRNAs exceeded 80%, and the number of downregulated mRNAs also accounted for more than half. Target genes were widely involved in immune-related biological processes (Figure 9(a)), which indicated that suppression of immune function in STC rats was mediated by lncRNAs.

Based on the absolute Pearson correlation coefficient over 0.98 and *P* values less than 0.001, we identified 904 pairs of coexpressed lncRNAs and mRNAs; all of them showed strong positive correlations (Figure 9(b)). Ranked by connectivity with mRNAs, the top 10 core regulatory lncRNAs identified were MSTRG.17256.1 (gene: LOC689757, degree = 101), ENSRNOT00000082578 (gene: AABR07025023.1, degree = 101), MSTRG.17262.1 (gene: Clec2d2, degree = 87), ENSRNOT00000092116 (gene: LOC102549869, degree = 84), MSTRG.17262.2 (gene: Clec2d2, degree = 82), ENSRNOT00000092808 (gene: AABR07035470.1, degree = 76), ENSRNOT00000093154 (gene: Fcrl5, degree = 75), MSTRG.4246.1 (gene: Mir142, degree = 74), ENSRNOT00000065330 (gene: AABR07044416.1, degree = 40), and MSTRG.10022.10 (gene: Cmahp, degree = 37). Similar to *cis* action, the relationships in the *trans*-regulatory network were mostly in the state of cosuppression, and GO annotations showed that the vast majority of the *trans*-target mRNAs are involved in the regulation process of immune signal and the proliferation and differentiation of immune cells (Figure 9(a)). Interestingly, all the core lncRNAs and their coexpression mRNAs in this network are downregulated (Figure 9(b)). It suggested that these lncRNAs might be the key factors to reduce the immunity.

### 3.11. CeRNA Network Analysis

circRNAs generally act as the sponge of miRNAs. By competing with mRNAs in a sequence complementary manner to binding miRNAs, circRNAs relieve miRNAs from inhibiting mRNAs translation, thereby exerting regulatory functions on protein coding genes [17, 18]. Therefore, we predicted the circRNAs-miRNAs-circRNAs binding relationship and constructed networks between DE circRNAs and DE mRNAs that were identified in this study.

**Figure 10:**
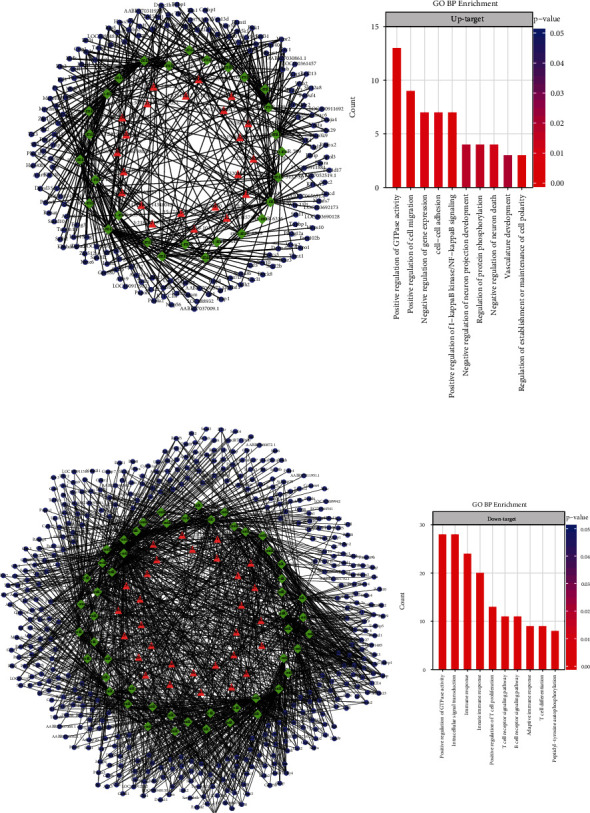
Analysis of DE mRNAs regulated by DE circRNAs. The ceRNA networks with high-score interactions of up- (a) or downregulated circRNAs (b). Triangles, diamonds, and circles represent circRNAs, miRNAs, and miRNAs, respectively. (c, d) Gene enrichment analysis for the host genes of target mRNAs for up- (c) or downregulated (d) circRNAs. Top 10 GO terms of biological process are shown, *y*-axis denotes the number of genes overlapping with each term, and color corresponds to the significance. Red and blue colors indicate upregulated and downregulated RNAs, respectively. The details about abbreviations of genes are listed in Table S1 and Table S2.

The co-activated network contained 22 circRNAs and 178 mRNAs, and the cosuppressed network contained 30 circRNAs and 425 mRNAs (Figures 10(a) and 10(b)). More than half of DE mRNAs had a potential regulatory relationship with circRNAs, indicating that circRNAs were generally involved in the regulation of related pathological processes. GO enrichment analysis of the target mRNAs in the coactivated network showed that many biological processes related to the nervous system and intercellular signal transduction were activated, such as regulation of establishment or maintenance of cell polarity, negative regulation of neuron death, negative regulation of neuron projection development, and cell-cell adhesion (Figure 10(c)). These functions were particularly more dominant than enriched functional of all upregulated mRNAs (Figure 10(a)), which implied that they were more regulated by circRNAs. A significant set of target mRNAs were related to immunoreaction (Figure 10(d)) in downregulated circRNAs, which was consistent with the overall trend of the functions for downregulating mRNAs or lncRNAs. It was shown that immune and inflammatory responses were universally regulated in STC rats.

## 4. Discussion

STC is an intestinal disease that affects the health and quality of life of patients. To reduce the incidence of STC, it is essential to understand the underlying pathophysiological mechanism. In this study, a group of differentially expressed transcripts and some key factors were identified that may regulate STC at the molecular level using a whole-transcriptome sequencing analysis in the colon tissue of STC rats.

There are larger amounts of deregulated mRNAs in STC rats than normal rats (Figure 9). According to enrichment analysis, the downregulated prominent functions are immune cell receptor and inflammatory pathways with its related signaling pathways (Figure 10(b)). Previous studies have shown that inflammation is closely related to gastrointestinal motility disorders; moreover, the release of inflammatory mediators leads to changes in gastrointestinal motility [19]. Our results are consistent with this, suggesting that inflammation may play an important role in STC progression. Interestingly, overexpressed mRNAs are also involved in immune responses (Figure 10(a)). Disorders of energy metabolism strongly affect the homeostasis of intestinal environment, which can accelerate a series of diseases progression such as intestinal inflammation and neoplastic pathology [20]. The release of inflammatory mediators can induce acute inflammatory cell infiltration and promote NF-*κ*B activity [21, 22], related pathways, which can be observed from upregulated mRNAs. The mRNAs included positive regulation of GTPase activity, cell migration and I-kappaB kinase/NF-kappaB signaling, phagosome, and glycerolipid metabolism (Figure 10 A).

Constipation symptoms are also inseparable with enteric nervous system dysfunction and changes of neurotransmitters that regulate intestinal motility [23]. In our study, the nervous system development, immunological synapse, and various cell receptor signaling pathway are downregulated (Figure 10). Interstitial cells of Cajal (ICC) are involved in intestinal neuromuscular signal transmission, which play an important role in regulating intestinal smooth muscle contraction and controlling gastrointestinal tract movements. Loss and dysfunction of ICC related to inflammation are the causes of STC [24–26].

In addition, we observed a large number of transcripts, which are related to encoding tumor necrosis factor ligands (Tnfsf8, Tnfsf9, and Tnfsf11) and receptors (Tnfrsf13c, Tnfrsf26, and Tnfrsf9), are downregulated in STC rats (Table S1). Tumor necrosis factor alpha (TNF*α*) and its related transcripts, usually acting as pathogenic proinflammatory cytokines, have been shown imbalanced in inflammatory intestinal tissues and peripheral lymphocytes [27, 28]. TNF-*α* is also known to be a particularly toxic agent to mitochondria, render whose dysfunction, and lead to cytokine storm of inflammatory activity [29]. Therefore, it is entirely plausible that the downregulation of TNF-*α*-related transcripts through affecting the mitochondrial function to shift energy production and activity of the inflammatory tissue in STC.

Among PPI network, the hub proteins are encoding products from vital mRNAs, and they were speculated to be important factors regulating STC (Figure 8). Dync1h1 encodes a member of the dynein cytoplasmic heavy chain family; its dysfunction can cause gut dysmotility [30]. Cd19 is a B cell coreceptor that is important for B cell development; its low expression can negatively affect the intestinal physiology under steady-state conditions [31]. Hsp90aa1 functions as a molecular chaperone and assists in the assembly, folding, and degradation of target proteins. The loss of Hsp90aa1 function is often accompanied by inflammation and other diseases [32]. Fn1 encodes fibronectin, and overexpression can cause fibrosis in various organs or tissues [33]. It is speculated that Fn1 may reduce the efficiency of intestinal transit and increase the risk of STC.

There are thousands of noncoding RNAs in human genome, which are widely involved in biological processes, such as imprinting and chromosomal conformation [34], coordination of cell status and differentiation [35, 36], enzymatic regulation [37], and disease [38]. Thus, the dysregulation of noncoding RNAs is closely related to diseases. However, there are still many gaps between the current understanding of the expression pattern and molecular process of noncoding RNAs in a specific gut environment. In our research, we use rats as animal models and focused on the regulation of two important noncoding RNAs, lncRNAs, and circRNAs.

Firstly, we annotate all expressed transcripts at the whole transcriptome level and describe their characterization (Figure 4). Nearly 90% of the coding genes are already recorded in related database, while half of the lncRNAs are recently identified. It indicated that there are still many unknown noncoding functional regions that need to be elucidated in the rat genome. Transcriptome-wide studies have shown that the expression of noncoding RNAs is usually more specific and strictly regulated than protein coding genes [39]. Thus, we speculate that these novel transcripts may be somehow related to disease process and partially specifically expressed in STC. Overall, our results further provided reference insights for the transcript expression of rats under STC status and enriched the rat genome resources.

The complex interaction of noncoding RNAs and coding genes are widely involved in the regulation of intestinal homeostasis and physiological processes [40]. By predicting the regulatory network of lncRNAs or circRNAs for coding genes, we observed many noncoding RNAs are involved in the pathological process of STC (Figures 9 and 10). The most prominent functions of target genes are immune and inflammatory responses, followed by the nervous system and intercellular signal transduction. This is consistent with the overall function of mRNAs, suggesting that a considerable portion of expression of the coding genes is regulated by noncoding RNAs, both at the transcriptional and posttranscriptional levels. Among networks, a group of key noncoding RNAs were identified. They are the core regulator that has more potential coding gene targets.

The current study illuminated the expression profiles of mRNAs, lncRNAs, and circRNAs in the loperamide-induced constipation in rats. We identified mRNAs, lncRNAs, and circRNAs with differential expression between the Lop group and the control group and elucidated the characteristics of mRNAs, DE lncRNAs, and regulatory functions of DE mRNAs. Besides, we tapped several core regulators that may contribute to the maintenance of intestinal transit. Our findings may provide useful insights into the molecular mechanisms underlying the development of STC. Further research is required to investigate the functions of mRNA, lncRNA, and circRNA identified in the present study.

## Figures and Tables

**Table 1:** Summary of the transcriptome sequencing data obtained in this study.

Sample name	Raw reads	Clean reads	Clean bases	Q20 (%)	Q30 (%)	GC content (%)	Total mapped	Uniquely mapped
K1	54767655	54434998	16.28G	98.75	96.31	52.40	51860222 (95.27%)	38980372 (71.61%)
K2	61081489	60695690	18.14G	98.75	96.34	52.41	57472748 (94.69%)	43010664 (70.86%)
K3	55462744	55116062	16.48G	98.65	96.00	49.77	51924842 (94.21%)	42681157 (77.44%)
K4	58025418	57717793	17.26G	98.78	96.34	50.45	54601032 (94.60%)	44730631 (77.50%)
M1	51348971	51116085	15.29G	98.76	96.29	53.18	48953874 (95.77%)	35627388 (69.70%)
M2	54848044	54544541	16.31G	98.66	96.07	53.36	51937311 (95.22%)	36996014 (67.83%)
M3	56249126	55882830	16.70G	98.74	96.30	52.31	53239572 (95.27%)	40143889 (71.84%)
M4	63916137	63522648	19.02G	98.83	96.56	53.09	60149595 (94.69%)	43119163 (67.88%)
M5	57250994	56608274	16.85G	98.74	96.43	53.44	53981650 (95.36%)	38764676 (68.48%)
Average	56994508	56626546	16.99G	98.74	96.3	52.27	53791205 (94.99%)	40450439 (71.43%)

**Table 2:** Top 7 hub proteins in protein-protein interaction network.

DE mRNAs	Express	Degree	Pathway
Dync1h1	Up	13	Immune system, neutrophil degranulation, cell cycle
Cd19	Down	9	Diseases of signal transduction, B cell receptor signaling pathway
Ptprc	Down	9	B cell receptor signaling pathway, T cell receptor signaling pathway, neutrophil degranulation
Hsp90aa1	Down	9	Diseases of signal transduction, G2/M transition
Dync1i2	Up	8	Golgi-to-ER retrograde transport, G2/M transition, cell cycle
Ccr7	Down	8	Chemokine superfamily pathway, Akt signaling
Fn1	Up	8	ERK signaling, MAPK signaling, integrin pathway

## Data Availability

The authors declare that the data supporting the findings of this study are available within the article and its Supplementary Information File or from the corresponding authors upon request.

## Supplementary Materials

Supplementary 1 Table S1: number of RNA-sequencing reads and mapping results. GO and KEGG results of DE mRNA, lncRNA, and circRNAs in the Lop and normal rats.

Supplementary 2 Table S2: a total of 109 possible *cis*-regulatory relationships were identified.
